# The role of calcium and CaMKII in sleep

**DOI:** 10.3389/fnsys.2022.1059421

**Published:** 2022-12-22

**Authors:** Yuyang Wang, Yoichi Minami, Koji L. Ode, Hiroki R. Ueda

**Affiliations:** ^1^Department of Systems Pharmacology, Graduate School of Medicine, The University of Tokyo, Tokyo, Japan; ^2^Laboratory for Synthetic Biology, RIKEN Center for Biosystems Dynamics Research, Suita, Japan

**Keywords:** NREM sleep, calcium, CaMKII, phosphorylation, sleep-promoting kinase, Ca^2+^-dependent hyperpolarization

## Abstract

Sleep is an evolutionarily conserved phenotype shared by most of the animals on the planet. Prolonged wakefulness will result in increased sleep need or sleep pressure. However, its mechanisms remain elusive. Recent findings indicate that Ca^2+^ signaling, known to control diverse physiological functions, also regulates sleep. This review intends to summarize research advances in Ca^2+^ and Ca^2+^/calmodulin-dependent protein kinase II (CaMKII) in sleep regulation. Significant changes in sleep phenotype have been observed through calcium-related channels, receptors, and pumps. Mathematical modeling for neuronal firing patterns during NREM sleep suggests that these molecules compose a Ca^2+^-dependent hyperpolarization mechanism. The intracellular Ca^2+^ may then trigger sleep induction and maintenance through the activation of CaMKII, one of the sleep-promoting kinases. CaMKII and its multisite phosphorylation status may provide a link between transient calcium dynamics typically observed in neurons and sleep-wake dynamics observed on the long-time scale.

## 1 Introduction

Until the mid-20th century, it was not widely accepted that a simple inorganic ion, calcium (Ca^2+^), could regulate such important and diverse physiological functions, serving as a messenger for regulating specific inter- and intra-cellular signaling. Dr. Setsuro Ebashi (1922–2006) pioneered the demonstration that Ca^2+^ is important for muscle contraction, opening the door for research on Ca^2+^ as a signaling molecule in the body (Endo, 2011). To date, Ca^2+^ is well recognized as one of the most commonly used cellular signaling molecules, acting in signal transduction *via* membrane proteins [e.g., N-methyl-D-aspartate receptors (NMDARs), voltage-gated calcium channels (VGCCs)] or intra-cellular proteins such as protein kinase PKC and CaMKII. Ca^2+^ plays an important role in signal transduction pathways, acting as second messenger in neurotransmitter release from neurons (Berridge, 1998), in the contraction of the muscle (Baylor and Hollingworth, 2011), and in fertilization (Kashir et al., 2013).

Calcium is associated with various temporal dynamics in life. In neurons, calcium changes neuronal activity on the order of milliseconds. Calcium waves play an important role in egg fertilization on the order of seconds (Kashir et al., 2013). In cardiomyocytes, Ca^2+^ is involved in the oscillation mechanism in heartbeats on the order of seconds (Eisner et al., 2017). Calcium is important in synaptic plasticity and it contributes to learning and memory formation on the order of hours (Mateos-Aparicio and Rodriguez-Moreno, 2020). Intracellular Ca^2+^ has diurnal variations in the suprachiasmatic nucleus (SCN) of the clock center (Enoki et al., 2012), and it is suggested that Ca^2+^ is important in the formation of circadian rhythms that create a daily activity rhythmicity in the body.

Of note, sleep is an evolutionarily conserved phenotype shared by most of the animals with nervous systems on the planet (Joiner, 2016). In humans, recent studies on sleep dysfunction have linked it to many psychiatric disorders (Anderson et al., 2005; Soehner et al., 2013; Reeve et al., 2015), including schizophrenia (Chan et al., 2017; Manoach and Stickgold, 2019) and bipolar disease (McKenna and Eyler, 2012). The molecular mechanisms of mammalian sleep regulation are still largely unknown. Recent mice genetic studies have given us more insight into genes and molecules that regulate animals’ sleep behavior. For example, a forward genetic screening in randomly mutagenized mice identified novel sleep-regulating genes in mammals (Funato et al., 2016). We also conducted reverse genetics approaches to identify sleep-regulating genes including Ca^2+^/calmodulin-dependent protein kinase II (CaMKII) and other genes controlling intracellular Ca^2+^ dynamics (Tatsuki et al., 2016). This review describes the forms of action and physiological functions of calcium and CaMKII in the different dimensions of sleep control as well as challenges of current research and future possibilities.

## 2 Sleep in various biological layers

### 2.1 Sleep at the behavioral level

**FIGURE 1:**
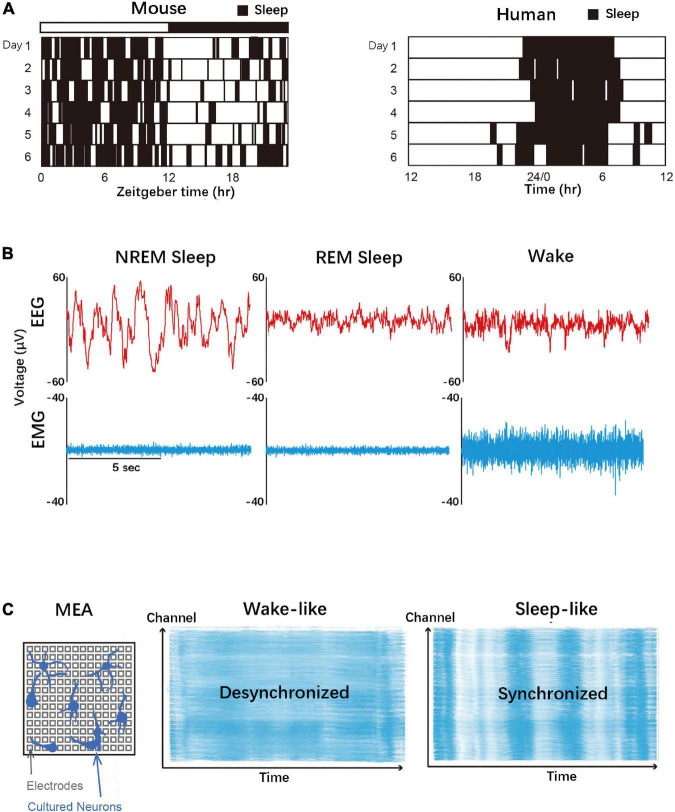
Sleep in various biological layers. **(A)** Mice and human sleep patterns. Mice are nocturnal animals and show polyphasic sleep, whereas humans are diurnal animals and show monophasic sleep. **(B)** Typical EEG/EMG recordings of wake, REM sleep, and NREM sleep in the adult human. **(C)** Schematic diagram of a microelectrode array (MEA) that can measure neuronal firing *in vitro*. The “wake-like (desynchronized)” and “sleep-like (synchronized)” patterns were observed depending on the culture conditions.

Sleep seems to be a phenomenon with ancient evolutionary roots. Sleep is a conserved phenotype within animal species (Joiner, 2016). All mammals with nervous systems, birds, and reptiles seem to sleep. Of all, except humans, mice remain the most commonly used model for sleep studies (Figure 1A). Experiments corresponding to sleep studies in mice are well established, e.g., knockout (KO) mice models for specific sleep genes. Sleep could be characterized by reduced movement, altered consciousness, and decreased responsiveness to external stimuli (Zielinski et al., 2016). Since this is a behavioral definition, it can be applied to animals other than mammals–fish and invertebrates also show sleep-like states. The jellyfish *Cassiopea* and *Hydra Vulgaris* are some of the most primitive organisms for which sleep-like behavior has been observed (Nath et al., 2017; Kanaya et al., 2020). The rate that jellyfish twitch their tentacles decreases during the night, and the animals’ response to external stimuli slows down during this period (Nath et al., 2017). Zebrafish showed high activity during the day, and sleep-like behaviors such as inactivity and caudal fin drooping were observed at night (Yokogawa et al., 2007). These small translucent fish contribute to sleep studies; they have the advantage of having a central nervous system (CNS) organization similar to that of mammals. *C. elegans* also shows sleep-like behavior of reduced responsiveness and reversibility during lethargus period (Raizen et al., 2008). *Drosophila melanogaster* also participated in sleep studies with similar characteristics as mammals (Huber et al., 2004b). The required sleep varies between species, ranging from giraffes and horses that sleep only 3 h to bats that sleep 18 h throughout the day (Siegel, 2005).

### 2.2 Sleep at the brain level

In mammals, the sleep-wake state can be referred to as peculiar brainwave patterns (Zielinski et al., 2016). Brain rhythms, which refer to the massed neuronal activity that can be monitored from the surface of the scalp typically by using electroencephalogram (EEG) (Figure 1B). During wakefulness, cortical neurons are hardly synchronized, presenting mainly high frequency and low amplitude beta waves (15–30 Hz) and gamma waves (30–90 Hz). Mammalian sleep can be divided into rapid eye movement (REM) state and non-rapid eye movement (NREM) state. NREM sleep is characterized by low frequency, large amplitude EEG rhythms presumably originated from the synchronous firing of cortical neurons. In humans, NREM sleep can be further classified according to the brain waves, including theta waves (3–7 Hz) in stage 1 followed by K-complex (<1 Hz) in stage 2. Sleep spindles (8–14 Hz) are also observed in stage 2, occasionally grouped with K-complex. Stage 3 in NREM sleep are clearly defined delta waves (1–4 Hz) and slow waves (<1 Hz) (Patel et al., 2022). On the other hand, EEG of REM sleep is more like wakefulness, with high frequency and low amplitude EEG patterns. Note that the presence of NREM and/or REM sleep is not limited to mammals but it is widely conserved across the animal kingdom (Tobler and Borbély, 1988; Yokogawa et al., 2007; Shein-Idelson et al., 2016).

Electroencephalogram slow wave activity (0.5–4 Hz, SWA) during NREM correlates with the expected levels of sleep need that is well explained by using two process model (Borbély et al., 1981). Process C or the circadian process, controls the timing of sleep and wakefulness during the day-night cycle, whereas Process S, or the sleep-wake homeostasis, represents the sleep need, which increases in proportion to the quantity and quality of the preceding awake state and decreases in the subsequent sleep state. Although the two-process model supposes Process C and Process S to act independently, the circadian clock and sleep needs may have close interactions at the cellular and molecular levels (Krueger, 2020). About the circadian clock systems, see previous reviews (Takahashi, 2017; Partch, 2020).

### 2.3 Local sleep in the cortical areas

Sleep and wakefulness may not be absolutely segregated but may be controlled heterogeneously among the cortical area. Part of the brain forced to awake for prolonged amounts of time may take a “local sleep” in a use-dependent manner (Krueger et al., 2008). Indeed, the level of SWA in the cortex can be heterogeneous depending on the level of neural activity during the preceding awake period (Huber et al., 2006). Researchers have also found that the animals appear to be awake with eyes open, and their EEG is wake-like, with only a subset of cortical neurons temporarily going offline and firing like slow wave sleep (SWS) (Huber et al., 2006; Vyazovskiy et al., 2011). In contrast, all the cortical neurons have large and slow rhythms with typical sleep EEG patterns, and the animals are relatively unresponsive in real sleep. They also discovered that prolonged wakefulness would increase these temporary off periods and was found to be correlated with worsened task performance in rats (Vyazovskiy et al., 2011). Interestingly, dolphins, whales, and some birds were found to sleep only one single cerebral hemisphere at a time so that they can stay alert in the demanding environment (Mukhametov et al., 1985; Lyamin et al., 2004, 2017). Some animals, including humans, also show microsleep (MS), which is defined as temporary sleep or drowsiness that lasts for seconds. Different from local sleep, individuals are unconscious and fail to respond to the external stimuli in MS, showing behaviors like nodding and droopy eyelids, etc. (Poudel et al., 2014). MS often results from sleep deprivation or monotonous tasks and is extremely dangerous if the job requires alertness, for example, driving (Innes et al., 2010; Poudel et al., 2014).

### 2.4 Sleep in cortical neurons

The neural circuits that produce sleep are not yet fully understood (Weber and Dan, 2016). Previously, people believed that the ascending reticular activating system (ARAS) promotes wakefulness while the ventrolateral pre-optic nucleus VLPO promotes sleep (Saper et al., 2005). With the research efforts done within these 15 years, however, it is still unknown whether these circuits connecting brain nuclei play a central role in controlling sleep need, as animals with disrupted ARAS still require a similar amount of sleep per day (Denoyer et al., 1991; Gerashchenko et al., 2004; Blanco-Centurion et al., 2007). In recent years, there has been an increasing number of discoveries of sleep-promoting and wake-promoting neurons (Weber and Dan, 2016; Liu and Dan, 2019; Jacobson et al., 2022). The REM and NREM sleep neurons constitute highly distributed networks spanning the forebrain, midbrain, and hindbrain. Liu and Dan proposed an arousal-action circuit for sleep-wake control in which wakefulness is supported by separate arousal and action neurons, while REM and NREM sleep neurons are part of the central somatic and autonomic motor circuits (Liu and Dan, 2019). Orexins (hypocretins) are peptide hormones produced exclusively in the lateral hypothalamus. Orexin receptors are widely expressed across the brain and participate in various neurophysiological functions, including sleep and wake, reward, fear, anxiety, and cognition, etc. And they are associated with enhanced wakefulness and activity (Jacobson et al., 2022).

Adamantidis et al. explain that there are three models of sleep-wake states: the flip-flop “switch” model, the reciprocal interaction model, and the thalamocortical loop model (Adamantidis et al., 2019). According to the former two models, sleep is defined as neural circuits. However, in the thalamocortical loop model, sleep is initiated locally at the neuronal levels. In this model, thalamic neurons have intrinsic electrical properties that regulate sleep and are not just a mediator that relays signals (Adamantidis et al., 2019). Therefore, more evidence could possibly be provided with the study of cortical neurons.

Local and heterogenous SWA in the cortex implies that cortical neurons contain essential properties, at least in part, for the homeostatic regulation of sleep. Synchronous firing of cortical neurons is thought to be the source of SWA in NREM EEG. During NREM sleep, cortical neurons exhibit a characteristic firing pattern, where the upstate with neuron bursting and downstate without bursting repeatedly appears (Steriade et al., 1993; Steriade et al., 2001). The frequency of up-down state switching is well matched with the slower (<1 Hz) component of SWA, while the 1–4 Hz component may be originated from thalamic and cortical oscillations (Amzica and Steriade, 1998). Such up-down state oscillation associated with sleep need is also observed in *Drosophila melanogaster*, implying that the oscillation is a conserved sleep feature across phyla (Raccuglia et al., 2019). The synchronous up-down state oscillation appears in the cortical cell population without inputs from the other brain area (Hinard et al., 2012).

SNAP25 (synaptosomal-associated protein 25kDa) is a t-SNARE protein responsible for synaptic vesicle fusion. Selective ablation of SNAP25 in neocortical layer V disrupts calcium-related neurotransmitter release and silences the neurons, which significantly increases wakefulness and reduces SWA rebound after sleep deprivation (Krone et al., 2021). A subset of cortical GABAergic interneurons expressing neuronal nitric oxide synthase (nNOS) might be involved in the propagation of slow waves through the cortex, along with several neuropeptides and neurochemicals participating (Schwartz and Kilduff, 2015). More specifically, cortical neurons that co-express nNOS and neurokinin-1 receptor (NK1R) have been found to play a critical role in sleep homeostasis, including NREM sleep time and NREM bout duration (Morairty et al., 2013).

Studies have further found that synchronous neural firing, similar to what is observed in an animal’s intact brain during sleep, occurs in isolated cortical neurons (Hinard et al., 2012; Jewett et al., 2015; Colombi et al., 2016; Saberi-Moghadam et al., 2018; Nguyen et al., 2019). Electrical or chemical stimulation of cultured neurons leads to an asynchronous, wake-like firing pattern, followed by a highly synchronous firing state that can be associated with rebound sleep response after sleep deprivation *in vivo*. These studies suggest that the sleep homeostatic mechanism works even in isolated neurons (Figure 1C).

### 2.5 Sleep on synapse

Homeostatic control or functions of sleep can be embedded in synapses. Synaptic plasticity is defined as the ability of neurons to respond to use or disuse by bringing about changes in the connections between neuronal networks (Hughes, 1958). Studies have found that molecules that relate to synaptic plasticity are actively expressed in regions during wakefulness, and the expression levels were significantly decreased during sleep (Cirelli and Tononi, 2000). Prolonged wakefulness was accompanied by difficulty in inducing LTP-like plasticity (Kuhn et al., 2016). Motor-learning tasks could induce an increase of SWA in subsequent sleep along with increased task performance (Huber et al., 2004a), while disruption of SWA in the motor cortex inhibited performance improvement (Fattinger et al., 2017). The exact relationship between SWA and LTP is still under discussion. A pioneering idea called the synaptic homeostasis hypothesis (SHY) of sleep proposes that during awake, which is optimal for learning, synapses are potentiated with increased synaptic strength. While asleep, slow-wave activity during NREM sleep induced downscaling and renormalization to preserve the synaptic strength at a conserved level (Tononi and Cirelli, 2003). Some findings are consistent with the model. *In vivo* recording also revealed cortical evoked responses increase during awake and decrease during sleep (Vyazovskiy et al., 2008). The size of synapses also changed with altered synaptic strength. During sleep, especially NREM sleep, is characterized by SWA, the size of synapse and the number of localized AMPARs decreases (de Vivo et al., 2017; Diering et al., 2017), which resembles LTD (Vyazovskiy et al., 2008). Meanwhile, there are some conflicting results. Post-learning sleep was reported to be critical for forming filopodia and spines in layer 5 pyramidal neurons in mice (Adler et al., 2021). Also, sleep deprivation decreased dendritic spine numbers in hippocampal CA1 but not in CA3 in mice (Havekes et al., 2016; Raven et al., 2019). In short, some papers support the SHY hypothesis that synaptic plasticity decreases during sleep, while others are inconsistent with the hypothesis. Sun et al. mentioned that changes in synaptic strength during sleep might vary according to brain regions (Sun et al., 2020).

## 3 Calcium in sleep

### 3.1 Theoretical analysis of neural activity during NREM sleep

Computational and theoretical biology has been a powerful tool to understand the molecular mechanisms underlying the cortical up-down state oscillation observed in SWS. Several models have been established to reconstruct SWS firing patterns (Timofeev et al., 2000; Hill and Tononi, 2005; Sanchez-Vives et al., 2010; Chen et al., 2012). In these studies, scientists attempt to reconstruct neuronal networks that include hundreds to thousands of neurons that reproduce the behavior of the cerebral cortex and thalamus (Compte et al., 2003; Hill and Tononi, 2005). The network model used by Compete et al. consists of 1,024 pyramidal neurons and 256 interneurons connected to each other by biologically rational synaptic dynamics, which also achieved the reproduction of slow wave oscillations (Compte et al., 2003). Hill and Tononi constructed a large-scale computer model that included parts of two visual areas, as well as the associated thalamic and reticular thalamic nuclei (Hill and Tononi, 2005). Their model assumed 28,880 model neurons in the primary visual area (Vp) layer and designed various connections between them in a computer, which successfully reproduced the slow-wave oscillations. Their studies share some interesting conclusions that the transition of the bursting phase and silent phase is mediated by some most common channels and receptors that mediate the transportation of Ca^2+^, Na^+^, and K^+^ (Compte et al., 2003; Hill and Tononi, 2005; Sanchez-Vives et al., 2010). However, the exact channels/receptors that participated are not yet clear. Previously mentioned models are dependent on the given parameters and require large-scale calculations, which is not suitable for the comprehensive exploration of possible parameter sets or pathways that are involved. Since previous studies have found that *ex vivo* cortical slices and even isolated neurons can reproduce slow wave oscillations (Sanchez-Vives and McCormick, 2000; Sanchez-Vives et al., 2010; Hinard et al., 2012; Colombi et al., 2016), inputs from other brain regions may not be necessary for SWA (Ode et al., 2017). Also, studies have found local sleep where some neurons in the cortex are temporarily shut off (Funk et al., 2016). Therefore, a dedicated neural network structure may not be required for the generation of cortical up-down state oscillation in SWS.

### 3.2 Calcium importance in sleep highlighted by “average neuron” model

In 2016, Tatsuki et al. put this idea into practice (Tatsuki et al., 2016). They first try to establish an “average neuron” using mean-field approximation, where the neuron could interact with itself directly or indirectly through excitatory or inhibitory synaptic currents (extrinsic currents). Aside from the extrinsic currents, intrinsic (non-synaptic) currents are also implemented based on previous models (Timofeev et al., 2000; Bazhenov et al., 2002; Hill and Tononi, 2005; Sanchez-Vives et al., 2010; Chen et al., 2012).

**FIGURE 2:**
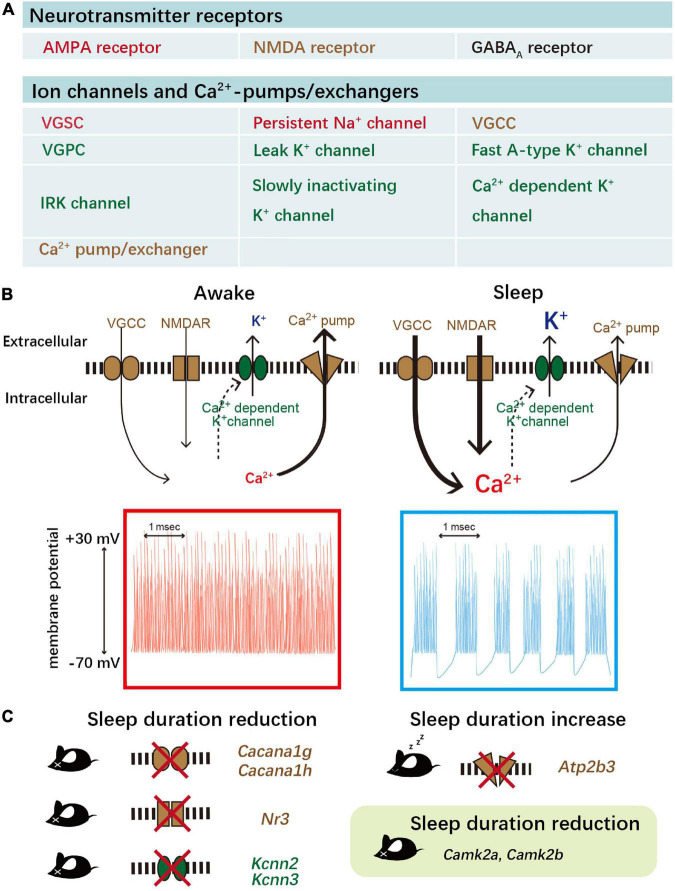
Ca^2+^-dependent hyperpolarization pathway neuronal firing during NREM sleep. **(A)** Neurotransmitter receptors, ion channels, and Ca^2+^ pumps/exchangers, which are involved in the “averaged neuron” (AN) model (Tatsuki et al., 2016). This model simplifies the neuronal network to average neurons by applying a rationale similar to the mean-field approximation. VGCC, voltage-gated calcium channels; VGSC, voltage-gated sodium channels; VGPC, voltage-gated potassium channels; IRK, inwardly rectifying K^+^ channels. **(B)** Ca^2+^ influx evokes SWS-like oscillations *in silico*. Ca^2+^ influx dominantly through Ca_v_ and NMDA receptors generates the bursting up-state in SWS-like oscillations, while K^+^ dominantly efflux through the K_Ca_ generates the down-state in SWS-like oscillations (right). Inhibition of Ca^2+^ influx or acceleration of Ca^2+^ efflux leads to awake-like firing without a down state (left). **(C)** Gene knockout studies demonstrated that ion channels/pumps suggested by the AN model play an important role in sleep regulations. Studies using mice also demonstrated that CaMKII α and β are important in the regulation of sleep duration.

More than 10,000,000 random parameter sets were generated, and 1,113 were selected that show SWS-like firing patterns. Bifurcation analysis was performed to understand which channels or pumps are of vital importance to the oscillation. Altogether, four candidates are proved to be important to generate SWS-like firing, which are NMDA receptors, voltage-gated Ca^2+^ channels (Ca_v_), Ca^2+^-dependent potassium channels (K_Ca_), and Ca^2+^ pumps. These four classes work in a calcium-dependent manner. In the up-state bursting phase, Ca^2+^ goes into the cell through NMDAR. Calcium influx depolarizes the membrane potential and further activates the Ca_v_. Accumulated Ca^2+^ activates the K_Ca_, and K^+^ effluxes the membrane to decrease the membrane potential to suppress the firing to go into the silent phase. Accumulated Ca^2+^ is exported through the Ca^2+^ pump. The suppression of calcium entry disappeared; thus, another firing could be observed. This Ca^2+^-dependent hyperpolarization pathway predicts that the impairment of the ion channels that allow calcium influx would decrease sleep duration, while impairment of the Ca^2+^ pump will increase sleep duration by facilitating hyperpolarization (Tatsuki et al., 2016). In this model, calcium accumulation is correlated with sleep duration (Figures 2A,B).

To explore the role of each gene involved in the Ca^2+^-dependent hyperpolarization pathway by generating gene knockout mice, we developed a CRISPR/Cas9-based high-throughput gene modification system called the triple-CRISPR technique (Sunagawa et al., 2016). The technique allows scientists to generate gene KO mice within 4 months. Sleep phenotypes were monitored using the respiration-based automated sleep phenotyping system Snappy Sleep Stager (SSS), which allows sleep phenotyping on a large scale (Sunagawa et al., 2016). Using these systems, we successfully disrupted all 29 genes that were involved in the AN model (Sunagawa et al., 2016; Tatsuki et al., 2016). KO mice with impaired K_Ca_ showed a decreased total sleep duration and also NREM sleep (SWS) duration, with a lower transition probability from an awake state to sleep (P_ws)_. K_Ca_ KO mice also have sleep homeostasis dysfunction after sleep deprivation (SD), with no significant SWS increase after SD. A study using local cortical depletion of K_Ca_ channels showed that K_Ca_ channels are important for the induction of SWA in the local cortex (Muheim et al., 2019). Then α1 subunits of Ca_v_, which determine the channel’s responsiveness to membrane depolarization, are also knocked out, and KO of Ca_v_3.1 and Ca_v_3.2 showed significantly decreased sleep duration, which is consistent with the previous findings (Lee et al., 2004; Anderson et al., 2005). The NMDAR subfamilies include the GluN1 (Nr1) subunit, four distinct GluN2 (Nr2) subunits, and two GluN3 (Nr3) subunits. *Nr3* KO mice exhibit a significant short-sleeper phenotype (Sunagawa et al., 2016). KO mice of *Nr1* or *Nr2b* exhibit lethal phenotypes (Forrest et al., 1994; Li et al., 1994) and were blocked pharmacologically by NMDAR antagonist MK-801 or phencyclidine (PCP). NMDAR perturbed mice showed a dose-dependent decrease in total sleep duration and also NREM sleep (SWS) duration compared to the controls. Blockade of NMDAR using MK-801 also increased neuronal excitability as measured by whole-brain imaging (Tatsuki et al., 2016). On the other hand, mice with impaired Ca^2+^ pumps decrease calcium efflux and increase sleep duration significantly. These results support that the Ca^2+^-dependent hyperpolarization pathway controls the sleep duration in mice (Figure 2C).

### 3.3 Evidence of calcium importance in sleep

Sleep researches other than mice have also highlighted calcium’s importance in sleep. *Drosophila melanogaster*, or fruit fly, has been widely used in biological research in the last century owing to its simple genetics and fast reproduction speed. Over the past decades, sleep studies on flies have led to gradual findings on genes and neuronal circuits that regulate sleep (Hendricks et al., 2000; Shaw et al., 2000; Lee et al., 2004). In line with our study, knockdown of the NMDA receptor gene (*Nmdar1*) or application of the NMDAR antagonist, MK-801, reduced sleep in flies (Tomita et al., 2015) which is consistent with the result in mice. Ca^2+^ influx through the NMDAR activates Calcineurin, a heterodimeric Ca^2+^/calmodulin-dependent serine/threonine protein phosphatase. Knockdown of calcineurin catalytic subunit A (*CanA1*), calcineurin regulatory subunit B (*CanB*), and calcineurin regulator *sarah* (*sra*) also results in a significant reduction in sleep (Nakai et al., 2011; Tomita et al., 2015). These results suggest that NMDAR-calcineurin signaling may play a role in sleep regulation. In 2016, Liu et al. found that elevated sleep pressure triggered reversible increases in cytosolic Ca^2+^ levels, NMDA receptor expression, and structural markers of synaptic strength (Liu et al., 2016).

T-Type Ca^2+^ channels are one of the low-voltage activated. They respond to small amplitudes and are inactivated while the cell membrane is hyperpolarized and then activated during depolarization. Ca^2+^ influx through the T-Type Ca^2+^ channels induces low-threshold spikes, which triggers the burst firing activity in corticothalamic neurons. T-Type Ca^2+^ channels have been implicated in sleep-related brain rhythms and the quantity and quality of sleep. Ca_V_3.1 KO mice showed a significant decrease in total sleep duration, resulting from a decrease in NREM sleep duration during the lighted period, and also accompanied by fragmented NREM sleep by brief wakefulness (Lee et al., 2004). Thalamic-specific Ca_V_3.1 KO also displays this NREM sleep fragmentation, which indicates the essential role of the thalamic T-type Ca^2+^ channel in blocking the transmission of arousal signals and stabilizing sleep (Anderson et al., 2005). An antagonist of the T-type Ca^2+^ channel was reported to enhance sleep (Yang et al., 2010). The same group reported another T-type Ca^2+^ channel antagonist, TTA-A2, in 2010, which suppresses active wake and promotes SWS in wild-type mice but not in mice lacking both Ca_v_3.1 and Ca_v_3.3 (Kraus et al., 2010). The T-Type Ca^2+^ channels are also linked to the brain waves associated with sleep. Ca_v_3.1 KO mice showed impaired delta oscillations, altered spindle oscillations, and intact slow oscillations (<1 Hz) (Lee et al., 2004). Meanwhile, Ca_v_3.3 mutant mice showed significantly decreased power density of spindle waves (Astori et al., 2011). The results suggest that Ca_v_3.1 plays an essential role in generating thalamocortical oscillations, and Ca_v_3.3 is responsible for the spindle oscillations.

Another type of Ca^2+^ channel, *Cacna1c* encoding for L-type voltage-dependent calcium channel, may be involved in sleep regulation. *Cacna1c* is a risk gene for bipolar and schizophrenia, characterized by sleep disturbance (Ament et al., 2015; O’Connell et al., 2019). Genetic knockout of the gene is lethal (Tatsuki et al., 2016), and heterozygous knockout of *Cacna1c* in mice induce reduced REM sleep recovery compared with their wild-type littermate (Kumar et al., 2015). A recent study indicates that it also plays a role in sleep regulation. Several *Cacna1c* variants are found to be associated with sleep latency (Kantojarvi et al., 2017).

### 3.4 Calcium in glial transmission

Astrocytes, a subtype of glial cells that tile the entire brain, may also play a key role in regulating sleep (Halassa et al., 2009; Pelluru et al., 2016; Clasadonte et al., 2017). Glial transmission is a phenomenon that describes the regulation of neuronal activity by astrocytes through the release of chemical transmitters. Inhibition of glial transmission reduced the accumulation of sleep stress and prevented the cognitive deficits associated with sleep loss (Halassa et al., 2009). Two-photon calcium imaging of neocortical astrocytes showed that an increase in calcium level in astrocytes precedes the spontaneous circuit shift to a slow-wave oscillatory state (Poskanzer and Yuste, 2016). Direct stimulation of astrocytes using optogenetic tools induced sleep (Pelluru et al., 2016) and switched the local neuronal circuit to this slow-oscillation state by altering extracellular glutamate (Poskanzer and Yuste, 2016). Recent studies have shown that astrocytic channel IP_3_-receptors (IP_3_Rs), which predominantly control the Ca^2+^ release from the endoplasmic reticulum (ER), are strongly implicated in sleep regulation. Transgenic overexpression of IP_3_-5-phosphatase mice in astrocytes attenuated IP3-mediated Ca^2+^ signaling in astrocytes and increased REM sleep and theta power (Foley et al., 2017). These findings suggest that IP_3_/Ca^2+^ signaling *via* IP_3_R is involved in the regulation of sleep. Although previous studies have focused on IP_3_R2, its KO in mice does not affect sleep (Cao et al., 2013). The active role of IP3-mediated Ca^2+^ signaling in astrocytes may also be supported by the observation that a general anesthetic at a lower concentration than inhibits neuronal responses is known to suppress IP3-mediated astrocyte Ca^2+^ responses (Thrane et al., 2012). In *Drosophila*, scientists detected Ca2 + levels within astrocytes vary with prolonged wake/sleep need using *in vivo* and *ex vivo* imaging (Blum et al., 2021). And sleep need is further transmitted to sleep drive circuit by releasing an interleukin-1 analog to act on the Toll receptor and R5 neurons (Blum et al., 2021).

Different neuronal and glial states in the sleep-wake cycle alter the extracellular ion environment, including Ca^2+^ (Ding et al., 2016). Extracellular K^+^ concentration is higher during the wake state, while extracellular Ca^2+^ and Mg^2+^ concentrations are higher during sleep. Such ion composition changes are not significantly affected by the inhibition of α-amino-3-hydroxy-5-methyl-4-isoxazolepropionic acid receptor (AMPA)-dependent synaptic inputs, but anesthesia reproduces the sleep-like extracellular ion conditions. Furthermore, it has been demonstrated that sleep and wakefulness can be induced by introducing artificial cerebrospinal fluid (ACSF) into the brain, which mimics the ionic environment during the sleep or wake phase, suggesting the causal role of extracellular ion conditions for the induction of sleep and awake neuronal firing patterns. Different extracellular ion conditions have been incorporated into mathematical models based on the AN model (Rasmussen et al., 2017). Mathematical analysis predicted that changes in the K^+^ conductance of K_Ca_ channels in response to changes in the extracellular ionic environment are important for the formation of slow-wave firing patterns. In addition, a large-scale computer model that reconstructs the circuit structure of cortical nerves predicts that an increase in extracellular Ca^2+^ concentration triggers synchronous firing of cortical nerves (Markram et al., 2015).

The extracellular ion concentrations are also altered between UP and DOWN states. The extracellular Ca^2+^ concentration increases toward the end of the DOWN state and decreases in the UP state (Massimini and Amzica, 2001). On the other hand, the extracellular K^+^ concentration increases in the UP state and decreases in the DOWN state (Amzica and Massimini, 2002; Amzica et al., 2002).

## 4 CaMKII in sleep

### 4.1 Phosphorylation hypothesis of sleep

**FIGURE 3:**
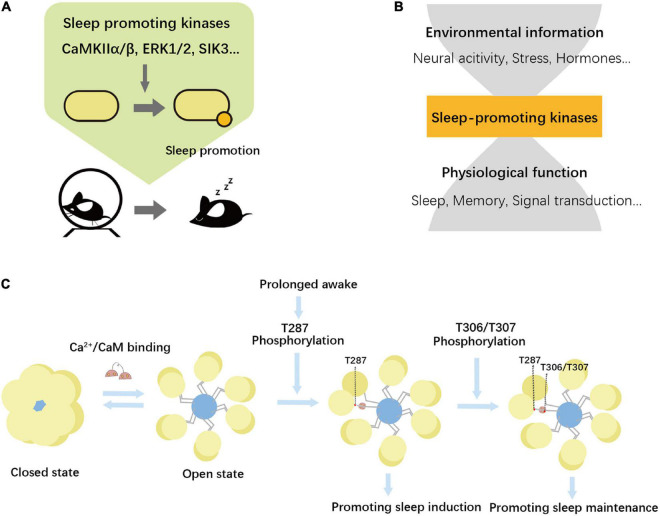
Sleep promoting kinases. **(A)** Genetic studies revealed sleep-promoting kinases, including CaMKIIα/β, SIK3, and ERK1/2. **(B)** Phosphorylation functions in many physiological phenomena. Environmental information and sleep-associated function could be integrated by sleep-promoting kinases. **(C)** CaMKII is a dodecameric protein complex with a ring-like structure. When the kinase is inhibited, CaMKII has a compacted structure. The compact inhibited state is under equilibrium with the open or extended active state. The active form is stabilized by the binding of the Ca^2+^/calmodulin (CaM) complex that allows for further autophosphorylation events. The residue number is based on CaMKIIβ. Details are shown in the text.

The role of the Ca^2+^-dependent hyperpolarization pathway in sleep implies that Ca^2+^ is also important for sleep serving as a signaling molecule inside the neurons, leading us to the finding of Ca^2+^/calmodulin-dependent protein kinase II (CaMKII) α and β as sleep-promoting kinases (Tatsuki et al., 2016). Physiological post-translational regulation of sleep-controlling protein appears to be associated with multiple kinase activities. Genetic and biochemical studies further revealed other potent sleep-promoting kinases, including salt-inducible kinase (SIK) 1/2/3 (Funato et al., 2016; Honda et al., 2018; Park et al., 2020), extracellular signal-regulated kinase (ERK) (Mikhail et al., 2017) (Figure 3A).

Funato et al. performed a large-scale forward genetic screening of more than 8,000 randomly mutagenized mice and identified a *Sleepy* mutant (Funato et al., 2016). Sleepy mice exhibited prolonged NREM sleep and increased NREM SWA. This particular phenotype was shown to result from a gain-of-function exon skipping of the salt-inducible kinase (SIK) 3. SIK3 is a member of the AMP-activated protein kinase (AMPK) family that is widely expressed in the brain. Of note, the exon skipping involves a phosphorylation site of SIK3, S551, targeted by protein kinase A (PKA) (Funato et al., 2016). S551A point mutation reproduced the NREM sleep promotion (Honda et al., 2018; Xu et al., 2022). Although the S551D mutant may not work for the phosphomimicking mutant and produced a similar sleep promotion effect, these results at least indicate that proper control of phosphorylation at S551 is important to the SIK3-mediated sleep regulation. The role of SIK3 in sleep promotion was conserved in *Drosophila* and *C. elegans*, indicating the conservation of sleep regulation in invertebrates (Funato et al., 2016).

Extracellular signal-regulated kinase (ERK) is another sleep-promoting kinase. The sleep-promoting activity of ERK was suggested in a study with *Drosophila*, where researchers found that sleep deprivation increases ERK phosphorylation in wild-type flies (Vanderheyden et al., 2013). Pan-neuronal expression of active ERK increased sleep, while ERK inhibitor reduced sleep (Vanderheyden et al., 2013). The role of ERK1/2 in the natural sleep-wake cycle was then confirmed in mice by embryonic and conditional knockout experiments (Mikhail et al., 2017). Also, *in vitro* studies showed that wake-like stimulation of primary cortical cultures could induce similar changes in gene expression as in sleep-deprived animals, including the overexpression of several serine-threonine protein kinases that inhibit MAPKs in ERK pathways (Hinard et al., 2012).

These and other kinases associated with sleep control may markedly affect the global phosphorylation status in neurons. A phosphoproteomics study revealed that among the quantified 4,500 proteins and ∼10,000 phosphopeptides in the post-synaptic density (PSD), about 20% of PSD proteins and phosphorylation sites showed changes in the expression level and/or phosphorylation level between day and night (Diering et al., 2017). Another phosphoproteomics study quantitatively compares the phosphorylation profile between mice with sleep deprivation and the *Sleepy* mutant mice, both of which should have a higher sleep need and elevated SWA (Wang et al., 2018). The study revealed the similarity of the phosphoproteomics profile between the two conditions against the untreated control mice. Furthermore, the study annotates 80 phosphoproteins whose phosphorylation levels are correlated with the expected amount of sleep need. The dominant role of the sleep-wake cycle for the phosphorylation level of synaptic proteins is further supported by the observation that sleep-wake cycles induce daily rhythms of many synaptic phosphoproteins, whereas sleep deprivation abolishes almost all phosphorylation cycles (Bruning et al., 2019). These findings lead us to propose the idea of the phosphorylation hypothesis of sleep, which assumes that sleep is induced by various kinases and such sleep-promoting kinases connect various signals to sleep-related physiological functions (Figure 3B).

### 4.2 CaMKII

Ca^2+^/calmodulin-dependent protein kinase II is widely expressed in the brain and is also a major downstream kinase of calcium signaling. It is known to interact with some of the candidates in the Ca^2+^-dependent hyperpolarization pathway. For example, CaMKII can directly bind to the NMDA receptor subunits NR1 and NR2B in response to Ca^2+^ influx and participate in LTP and memory (Leonard et al., 1999; Rose et al., 2009; Tao et al., 2021).

#### 4.2.1 Structural and functional properties of CaMKII

Ca^2+^/calmodulin-dependent protein kinase II forms a dodecameric complex with a ring-like structure and has a unique activity regulation through its autophosphorylation activity (Figure 3C). The kinase is made up of 12 subunits which together form the central organizing hub. Each subunit is composed of a catalytic kinase domain, regulatory segment, and central hub (Hoelz et al., 2003). When the kinase is auto-inhibited, CaMKII has a very compact structure. The regulatory segment blocks the substrate-binding site (S-site) and isolates the T286 (CaMKIIα) and T287 (CaMKIIβ) phosphorylation sites, which avoids calmodulin binding (Rosenberg et al., 2005). When Ca^2+^/calmodulin complex binds to CaMKII, the regulatory segment is stripped off and allows substrate binding and phosphorylation. The activated CaMKII phosphorylates T286/T287 through autophosphorylation, which allows the kinase to maintain an “autonomous” open active structure without Ca^2+^/calmodulin. However, CaMKII is not fully activated yet but could be further stimulated by Ca^2+^/CaM (Coultrap et al., 2010). CaMKII further autophosphorylates other CaMKII residues, including T305/T306 (CaMKIIα) and T306/T307 (CaMKIIβ), of which phosphorylation inhibits Ca^2+^/calmodulin complex binding abilities and vice versa (Colbran and Soderling, 1990; Hudmon and Schulman, 2002). The downstream substrate of CaMKII includes several neuronal proteins (e.g., GluN2B, GluA1, TIAM1) (Yasuda et al., 2022). These unique features of kinase modification offer the possibility that CaMKII maintains its kinase activity over a period of time. In *Drosophila*, the auto-regulatory activity of CaMKII acts as a timer within a time frame of a few minutes (Thornquist et al., 2020).

#### 4.2.2 CaMKII in sleep *in vivo*

Ca^2+^/calmodulin-dependent protein kinase II KOs are lethal in fruit flies and cause reproductive abnormalities in the second generation of mice. But scientists successfully produced homozygous *Camk2* KO mice using the triple CRISPR technique (Tatsuki et al., 2016). Embryonic knockout of *Camk2a* or *Camk2b* showed a significant decrease in sleep duration in mice (Tatsuki et al., 2016). Additionally, *Camk2a* or *Camk2b* knockout also reduced the transition probability between sleep and awake (Tatsuki et al., 2016). Consistent with the phenotype of these knockout mice, the post-natal expression of CaMKII kinase inhibitor AIP2 by the adeno-associated virus (AAV) vector resulted in a decrease in sleep duration (Tone et al., 2022). The reduced sleep duration of the AIP2-expressed mice, and at least for *Camk2b* knockout mice, was attributed to the reduced NREM sleep duration. Consistently, these mice showed an altered NREM delta-power profile (Tone et al., 2022).

The knockout and brain-wide inhibition of CaMKII activity demonstrate that CaMKII is a sleep-promoting kinase. However, we note that CaMKII has brain area-specific roles in the control of sleep. In brainstem pedunculopontine tegmentum (PPT) neurons, the phosphorylation level of CaMKIIα T286 is correlated with increased wakefulness (Stack et al., 2010), but the injection of CaMKII inhibitor KN-93 to the PPT decreased wakefulness, suggesting that in pedunculopontine tegmentum (PPT or PPN) neurons CaMKII is associated with promoting wakefulness (Datta et al., 2011; Urbano et al., 2014). Conversely, in the dorsal raphe nucleus (DRN), KN-93 microinjection suppressed wakefulness and enhanced NREM sleep and REM sleep (Cui et al., 2016). In the cortex and hippocampus in rats, the phosphorylation status of Thr286 in CaMKII but not the total protein level was also found to be higher in the waking group compared to the sleeping group (Vyazovskiy et al., 2008).

Phosphorylation states of CaMKIIα and CaMKIIβ are also affected by the sleep-wake cycle. Phosphoproteomics of PSD protein and western blotting of synaptosome showed that activated CaMKIIα with autophosphorylation at T286 is higher in the dark (active) phase. Interestingly, CaMKIIα with phosphorylated T310 is lower in the dark phase (Vyazovskiy et al., 2008; Diering et al., 2017), suggesting that CaMKIIα undergoes different phosphorylation states depending on the time of day and/or sleep-wake states. Western blot results also showed that CaMKIIα pT286 increases during wakefulness and decreases during sleep (Bruning et al., 2019). The changes in the phosphorylation of CaMKIIα T286 and CaMKIIβ T287 appeared to be correlated to the level of sleep need because these phosphorylation levels were increased by sleep deprivation (Wang et al., 2018; Tone et al., 2022).

The role of the phosphorylation status of CaMKII in sleep promotion is recently demonstrated by using AAV-mediated expression of CaMKIIβ and its phosphomimetic mutants (Tone et al., 2022). Through the comprehensive sleep phenotyping of mice expressing CaMKIIβ mutants, each of which had a phosphomimetic mutation at either of all serine or threonine residues, researchers found that phosphomimetic mutation at T287 (T287D) induced the increase in NREM sleep and SWA. This phenotype is opposite to what is observed in *Camk2b* knockout mice, further supporting the importance of CaMKII as a sleep-promoting kinase. CaMKIIβ having multiple phosphomimetic mutations at T306/T307 as well as T287 (T287D:T306D:T307D) also increases NREM sleep. Interestingly, however, the mode of sleep induction is qualitatively different between T287D-expression and T287D:T306D:T307D-expression. CaMKIIβ T287D expression increases the transition probability from awake to sleep, namely, the mutant promotes the sleep induction step. On the other hand, T287D:T306D:T307D-expression leads to a decrease in the transition probability from sleep to awake. Thus, this mutant promotes sleep maintenance (Tone et al., 2022). It has been suggested that phosphorylation of T305 and T306 (CaMKIIα) affects substrate specificity and neuronal localization of CaMKII and switches CaMKII’s contribution to neuronal plasticity from LTP to LTD (Coultrap et al., 2014). The multi-site and multi-step phosphorylation of CaMKII may also control the sleep-wake transition cycle at different steps. As discussed in this review, changes in neuronal firing patterns associated with the sleep-wake cycle led to different Ca^2+^ dynamics in neurons. On the other hand, multi-site autophosphorylation of CaMKII has been shown to be efficiently phosphorylated by Ca^2+^ signals of different frequencies (De Koninck and Schulman, 1998; Coultrap et al., 2014). Thus, neuronal Ca^2+^ dynamics and the regulation of sleep-wake transitions by CaMKII are probably closely interacting with each other.

There are four different CaMKII isoforms: α, β, δ, and γ that are different but highly homologous (Zalcman et al., 2018). The isoforms of CaMKII subunits differ in the linker sequence that connects the kinase domain to the central hub (Bhattacharyya et al., 2020). Notably, CaMKII can form homo- and hetero-polymeric holoenzymes with no known preference for isoforms (Bayer et al., 2002; Bayer and Schulman, 2019). Previous studies have revealed that when CaMKIIα and CaMKIIβ are expressed together, the two isoforms form heterooligomers. A small portion of CaMKIIβ is enough to dock the predominant CaMKIIα to the actin cytoskeleton, which supports the hypothesis that the primary function of CaMKIIβ is to alter the localization of CaMKIIα, first forming CaMKIIα/β heterooligomers and then localizing the hybrid complex to the new docking site (Shen et al., 1998). In sleep studies, whether and how CaMKIIα/β heterooligomers participate in sleep induction and maintenance is future work. Also, the different isoforms of CaMKII determine the phosphorylation outcomes (Bhattacharyya et al., 2020). CaMKIIα with a shorter linker tends to acquire activating autophosphorylation on T286, while CaMKIIβ with a longer residue linker is biased to be phosphorylated at T305/T306, which is the inhibitory autophosphorylation. Further studies unveil the secrets of CaMKII isoforms in sleep research.

## 5 Concluding remarks

In this review, we have discussed calcium, CaMKII, and sleep. An important discussion is about sleep in different layers. The sleep-like behavior found in primary cultured neurons suggests that organismal-level sleep behavior may be assembled from neuronal assemblies. Therefore, molecular studies may provide good evidence for the regulatory mechanisms of sleep homeostasis. The insight from the neuronal firing activity during NREM sleep highlighted the importance of calcium in sleep regulation, and mice studies supported the idea. Noteworthy, gene perturbation of *Camk2a/b* disrupted sleep. Phosphoproteomicsgen analysis revealed that protein phosphorylation levels, including CaMK2α/β, were dynamically changed according to sleep/wake status. Together with findings of SIK3 and ERK phosphorylation level changes, these findings support the idea of the phosphorylation hypothesis in which phosphorylation level changes of proteins are key features of sleep regulations. Because calcium is involved in almost all neural behaviors, what kind of calcium behaviors are recognized by specific sleep-controlling kinases such as CaMKII to regulate sleep-related functions and at which phosphorylation sites require further investigation. Therefore, future efforts could be devoted to identifying which phosphorylation sites are important and the corresponding sleep phenotypes.

## Author contributions

HU conceived, supervised the study, and all aspects of the work. YW, YM, and KO wrote the first draft of the manuscript and designed the figures. All authors contributed to the manuscript revision and approved the submitted version.
